# The Combination of Sonographic Features and the Seven-Gene Panel May be Useful in the Management of Thyroid Nodules With Indeterminate Cytology

**DOI:** 10.3389/fendo.2021.613727

**Published:** 2021-02-24

**Authors:** Marco Capezzone, Silvia Cantara, Andrea Di Santo, Alfonso Sagnella, Tania Pilli, Lucia Brilli, Cristina Ciuoli, Fabio Maino, Raffaella Forleo, Alessandra Cartocci, Maria Grazia Castagna

**Affiliations:** ^1^ Department of Medical, Surgical and Neurological Sciences, University of Siena, Siena, Italy; ^2^ Pathology Unit, University of Siena, Siena, Italy; ^3^ Department of Medical Biotechnologies, University of Siena, Siena, Italy

**Keywords:** thyroid nodules, thyroid ultrasound, fine needle aspiration cytology (FNAC), indeterminate nodules, molecular test

## Abstract

**Introduction:**

The management of patients with indeterminate thyroid nodules, which account for 10–25% of thyroid fine needle aspiration biopsies (FNABs), is still very challenging.

**Aim:**

To verify the utility of the seven-gene panel in combination with ultrasound features in the clinical management of indeterminate thyroid nodules.

**Results:**

The study group included 188 indeterminate thyroid nodules, divided into TIR3A (56.4%) and TIR3B (43.6%). A significant correlation between US categories and both cytological and molecular results was observed. In detail, TIR3B cytology was more frequent in EU-TIRADS 4 and 5 nodules (54.7 and 50%, respectively) than in EU-TIRADS 2 and 3 nodules (31%, *p* = 0.04). Similarly, the rate of a nodule with a mutation increased with the increase of US risk class (6.0% in EU-TIRADS 2 and 3, 9.3% in EUTIRADS-4 and 27.8% in EUTIRAD-5, *p* = 0.01). Among thyroid nodules submitted to surgery, final histology was benign in 61.4% nodules, while malignancy was diagnosed in 38.6% nodules. Using US score as tool for decision-making in TIR3A subgroup, we correctly classified 64.5% of thyroid nodules. The second tool (seven-gene panel test) was used in the subgroup of US high-risk nodules. By multiple tests with a series approach (US in all cases and US plus seven-gene panel in US high risk nodules) 84% of cases were correctly classified. In TIR3B nodules, using only seven-gene panel as tool for decision making, we correctly classified 61.9% of indeterminate nodules. By multiple tests with series approach (seven-gene panel in all cases and seven-gene panel plus US score in non-mutated nodules) only a slight improvement of thyroid nodule classification (66.6%) was observed.

**Conclusions:**

US score seems able to correctly discriminate between TIR3A nodules in which a conservative approach may be used, and those in which additional test, such as molecular test, may be indicated. On the contrary, in TIR3B nodules both US risk stratification and seven-gene panel seem to be of little use, because the risk of thyroid cancer remains high regardless of US score and mutational status.

## Introduction

The management of thyroid nodules cytologically defined as “indeterminate” is still very challenging. Therefore, it has been advocated to apply individualized strategies based on more accurate anatomo-pathological classifications and the use of immunocytochemical or molecular markers (1). The evaluation of molecular markers in the thyroid cytological specimens has been accepted in 2015 by the American Thyroid Association (ATA), as well as by other scientific societies, in the diagnostic work-up of indeterminate nodules (2, 3). The first molecular test was developed starting from the genetic alterations of the seven genes mostly involved in the papillary and follicular thyroid cancer, *i.e.*, point mutations within *BRAF, KRAS*, *HRAS* and *NRAS* genes, and *RET/PTC1*, *RET/PTC3* and *PAX8/PPARγ* rearrangements (4, 5). Of note, the tests were initially performed using molecular techniques of limited sensitivity (6). Although it has been previously reported that the seven-gene panel has high PPV and can “rule-in” cancer, it is likely that this tool has a relatively poor performance in this setting because of the low specificity of RAS mutations. Moreover, based on the low sensitivity, the seven-gene panel cannot “rule-out” cancer and a negative molecular result cannot avoid diagnostic surgery limiting their cost-effectiveness (7). The availability of molecular markers enhanced was significantly increased by the advent of next generation sequencing technology, and the gene expression classifier has also added a valuable contribution in the diagnosis of thyroid nodules (8, 9). However, these tools are not routinely used in Europe because of the high cost (10). Recently, the availability of high-frequency ultrasonography and the development of risk scores that can quantify the risk of malignancy have significantly implemented the decision-making in the thyroid nodules (11). Several risk-scoring systems have been developed which aim to reduce the inter-observer variability and to help the clinicians to make decisions regarding the work-up and follow-up of thyroid nodules (12–14). More recent studies suggest that repeating US risk stratification can be useful in predicting malignancy in the indeterminate nodules (15–17). However, in indeterminate thyroid nodules at high risk of malignancy, the utility of this approach may be limited (18).

Thus, we aimed to establish whether US features combined with the seven-gene panel results may significantly contribute to the pre-surgical diagnosis of nodules with indeterminate cytology.

## Patients and Methods

### Study Population

A retrospective analysis was conducted on 503 indeterminate thyroid nodules belonging to 467 patients with uni- or multinodular thyroid disease, who underwent fine-needle aspiration cytology (FNAC) at the Section of Endocrinology of the University of Siena, Italy, from 2009 to 2019. The study cohort included 119 males (25.5%) and 348 females (74.5%), with a mean age at diagnosis of 54.1 ± 14.1 years (range 16–93 years). In 73/188 (38.8%) indeterminate thyroid nodules, surgery was performed. Criteria for surgery were: 1) indeterminate nodule diameter >3 cm; 2) large size of uni- or multinodular goiter; 3) young age at diagnosis; 4) positive seven-gene panel.

### Methods

#### Ultrasound Stratification

All thyroid nodules were assessed by using a high-resolution ultrasound color Doppler apparatus (My Lab 40 HD, Esaote Biomedica, Firenze, Italy) with a 12 MHz linear transducer by two dedicated endocrinologists, who usually perform more than 1,000 neck ultrasound examinations per year. US reports and images of each thyroid nodule included in the study were recorded in the database by the endocrinologist who performed the examination. We classified the ultrasound reports based on the European Thyroid Association Guidelines for Ultrasound Malignancy Risk Stratification of Thyroid Nodules in Adults (EU-TIRADS) (14). Thyroid nodules were divided in four group: *EU-TIRADS 2 (benign category*); *EU-TIRADS 3 (low-risk category*); E*U-TIRADS 4 (intermediate-risk category*): *EU-TIRADS 5 (high-risk category*).

#### Cytological Evaluation

FNAC was performed by the same endocrinologists upon ultrasound guidance using a 23/25-gauge needle. The material was air dried, stained with May-Grunwald Giemsa and interpreted by one experienced cytologist who was blinded to the US risk of thyroid nodules. All cytological smears were reviewed by the same pathologist (DA). Thyroid nodules were cytologically defined as low-risk indeterminate (TIR3A) or as high-risk indeterminate (TIR3B) according to the Italian Society of Anatomic Pathology and Diagnostic Cytology (SIAPEC-IAP) (19). In detail, thyroid nodules that fall in the TIR3A category are characterized by increased cellularity, poor colloid content, and several but insufficient microfollicular structures to make the diagnosis of a “follicular neoplasia”. In these nodules, the expected risk of malignancy is lower than 10% suggesting that a follow-up with eventually a repetition of FNA within 6-months is adequate. Thyroid nodules with a TIR3B cytology are characterized by high cellularity arranged in monotonous microfollicular/trabecular structures, with poor/absent colloid (a picture suggestive of “follicular neoplasia”), and included also those cases with mild or focal nuclear atypia suggestive of papillary carcinoma and Hurthle cell neoplasms; the risk of malignancy in this category is between 15 and 30% and the surgical excision is considered the first-line of the therapeutic strategy.

#### Molecular Diagnosis

Since 2009, the Section of Endocrinology of the University of Siena has represented the Reference Center for the mutational analysis of thyroid nodules in the South-East of the Tuscany Region. The protocol for TIR3 nodules, adopted since then at our Institute, provides for the application of a seven-gene molecular test. Patients whose FNAC results were indeterminate (TIR3A/TIR3B) were subjected to a second FNA procedure, performed twice consecutively in the same session. All patients who submitted to FNA procedure signed informed consent. One sample was used for repeating cytological examination, and the other was dispersed into TRI Reagent buffer (Sigma) and stored at −20°C until DNA and RNA extraction. A specimen was qualified as satisfactory if there were six groups of epithelial cells with at least 10 cells per group. Smears were reviewed by the same pathologist and analyzed by a molecular biologist with the seven-gene panel searching for mutations. Samples were screened for the presence of BRAF (V600E and K601E), H- K-NRAS and hTERT mutations point mutations and for RET/PTC-1 and -3 and PAX8-PPARgamma rearrangements. For BRAF point mutations (V600E and K601E), exon 11 and exon 15 were amplified in a mixture containing 2× PCR Master Mix (AmpliTaq Gold^®^ PCR Master Mix, Applied Biosystems) and a final primer concentration of 200 nM at 52.5°C. To identify RAS mutations (H-, K-, and N-RAS) codons 12, 13 and 61 were amplified with 200 nM primer final concentration at 64.9°C for H-RAS and 58°C for K-RAS and N-RAS. hTERT was evaluated using a Fast PCR procedure at 63°C and in the presence of a GCrich solution (Roche). PCR products were subjected to direct sequencing. Rearrangements were evaluated with real time RT-PCR. For PAX8/PPAR*γ* 5-50 ng of cDNA was amplified in a final volume of 50 μl using 40 pmol of specific primers, 2 pmol of probes for 40 cycles at 72°C. Oligonuclotide sequences were: PAX8 (exon7)-F: 5’-AACCTCTCGACTCACCAGACCTA-3’, PAX8 (exon9)-F: 5’-CGGACAGGGCAGCTATGC-3’, PPAR*γ*-R: 5’-GTTGGTGGGCCAGAATGG-3’, PPAR*γ* Probe 5’-6FAM-CATGGTTGACACAGAGAT-MGBNFQ-3’. For RET/PTC-1 and -3, in a final volume of 20 µl we amplified 500 ng of cDNA in a mix containing 200 nM final concentration of specific primers and 100 nM of probes. Primers forward and probes were as follows: RET/PTC1 F 5’-CGCGACCTGCGCAAA-3’, RET/PTC3 F 5’-CCCCAGGACTGGCTTACCC-3’, PTC1 probe 5’-CAAGCGTAACCATCGAGGATCCAAA-3’, PTC3 probe 5’-AAAGCAGACCTTGGAGAACAGTCAG-3’. For both fragments, primer reverse was: RET/PTC R 5’-CAAGTTCTTCCGAGGGAATTCC-3’. Thermal cycling profile was 3 min at 95°C followed by 15 s at 95°C and 1 min at 60°C for 45 cycles.

#### Statistical Analysis

Epidemiological data are presented as the mean ± SD and median when needed. To evaluate significant differences in data frequency we analyzed contingency tables. Tables with size larger than 2 × 2 were examined by the Chi-squared test or a numerical approximation of the Fisher exact test when all cell frequencies were greater than 4 or not, respectively. “Test for trend” (X square for trend or Harmitage test) was also performed. Thyroid nodules were retrospectively classified according to EU-TIRADS system. For the statistical analysis, nodules classified as EU-TIRADS 2 and 3 or EU-TIRADS 4 and 5 were grouped separately, and thyroid nodules classified at histology as non-invasive follicular thyroid neoplasm with papillary like nuclear features (*NIFTP*) were classified as thyroid cancer. In order to improve the pre-surgical diagnosis of indeterminate thyroid nodules, a multiple test with a series approach was also used (20). In a series approach the initial test served as a screening function, and the latter test was ordered on a ‘‘pre-screened’’ population. In TIR3A nodules, thyroid US was used as screening test while a seven-gene panel test was used as second test but only in the subgroup of patients with US high risk thyroid nodules. Since this subgroup of indeterminate thyroid nodules has a low rate of cancer, we aimed to improve our ability in identifying benign nodules by using this approach. Conversely, in TIR3B thyroid nodules, seven-gene panel test was used as screening test, while thyroid US was used as a second test only in the subgroup of patients with a negative seven-gene panel. Since this subgroup of indeterminate thyroid nodules has a higher rate of cancer, we aimed to improve our ability in identifying malignant nodules by using a second test.

Statistical analysis was performed using the software StatView for Windows version 5.0.1 (SAS Institute, Cary, NC) and the *SPSS* Statistics version 22.0. A *p*-value <0.05 was considered statistically significant.

## Results

Among the 503 indeterminate thyroid nodules, cytological diagnosis of TIR3 was confirmed in 386/503 (76.7%) nodules. In the remaining 117/503 (23.3%) nodules, benign cytology (TIR2) was found at FNAC repetition, and they were excluded from the study. In the confirmed TIR3 nodules (n = 386), the seven-gene panel test was performed (point mutations within *BRAF, KRAS*, *HRAS*, and *NRAS* genes and *RET/PTC1*, *RET/PTC3* and *PAX8/PPARγ* rearrangements were searched) and molecular test was positive in 32/386 (8.3%) cases [five (15.7%) BRAF mutations, eight (25%) RET-PTC rearrangements, 16 (50%) RAS mutations, three (9.3%) PAX8/*PPARγ* rearrangements]. Since ultrasound and follow-up data were not available for all patients, the subsequent analyses were performed in a subgroup of 188 TIR3 thyroid nodules from 182 patients followed at the Section of Endocrinology of University of Siena.

### Cytological Series (n = 188)

As shown in **Table 1**, the study population included 42 males (22.3%) and 146 females (77.7%) with a median age of 55 years (range 18–88). Multinodular goiter was observed in 105/188 (55.8%) patients, and the median maximum diameter of indeterminate nodules was 20 mm (range 7–83). According to the European Thyroid risk stratification (14), the study group included 84 (44.7%) EU-TIRADS 2–3, 86 (45.7%) EU-TIRADS 4, and 18 (9.6%) EU-TIRADS 5 thyroid nodules. Among the 188 thyroid nodules with indeterminate cytology, 106 (56.4%) were TIR3A and 82 (43.6%) were TIR3B. Twent-six/82 (31.7%) of nodules in TIR3B category were Hurtle cell neoplasms. Using the seven-gene panel test, 18/188 (9.6%) nodules with a mutation were found. BRAFV600E was observed in two (11.1%) thyroid nodules, RAS mutations in 10 (55.6%), and RET/PTC rearrangement in six (33.3%).

**Table 1:** Demographic and clinical-pathological features in patients with indeterminate thyroid nodules (n = 188).

Parameters	TIR3 nodules (n=188)
**Gender: n (%)**	
Male	42 (22.3)
Female	146 (77.7)
**Age at diagnosis (years)**	
Mean ± SD	54.5 ± 14.0
Range	18–88
Median	55
**Baseline Nodule diameter (mm)**	
Mean ± SD	23.9 ± 12.1
Range	7–83
Median	20
**Type of goiter: n (%)**	
Multinodular goiter	105 (55.9)
Uninodular goiter	83 (44.1)
**Ecographic score: n (%)**	
EU-TIRADS 2/3	84 (44.7)
EU-TIRADS 4	86 (45.7)
EU-TIRADS 5	18 (9.6)
**Cytology: n (%)**	
TIR3A	106 (56.4)
TIR3B	82 (43.6)
**Presence of mutations: n (%)**	
yes	18 (9.6)
no	170 (90.4)
**Type of mutation: n (%)**	
BRAF	2 (11.1)
H-K-NRAS	10 (55.6)
RET/PTC	6 (33.3)

**Figure 1:**
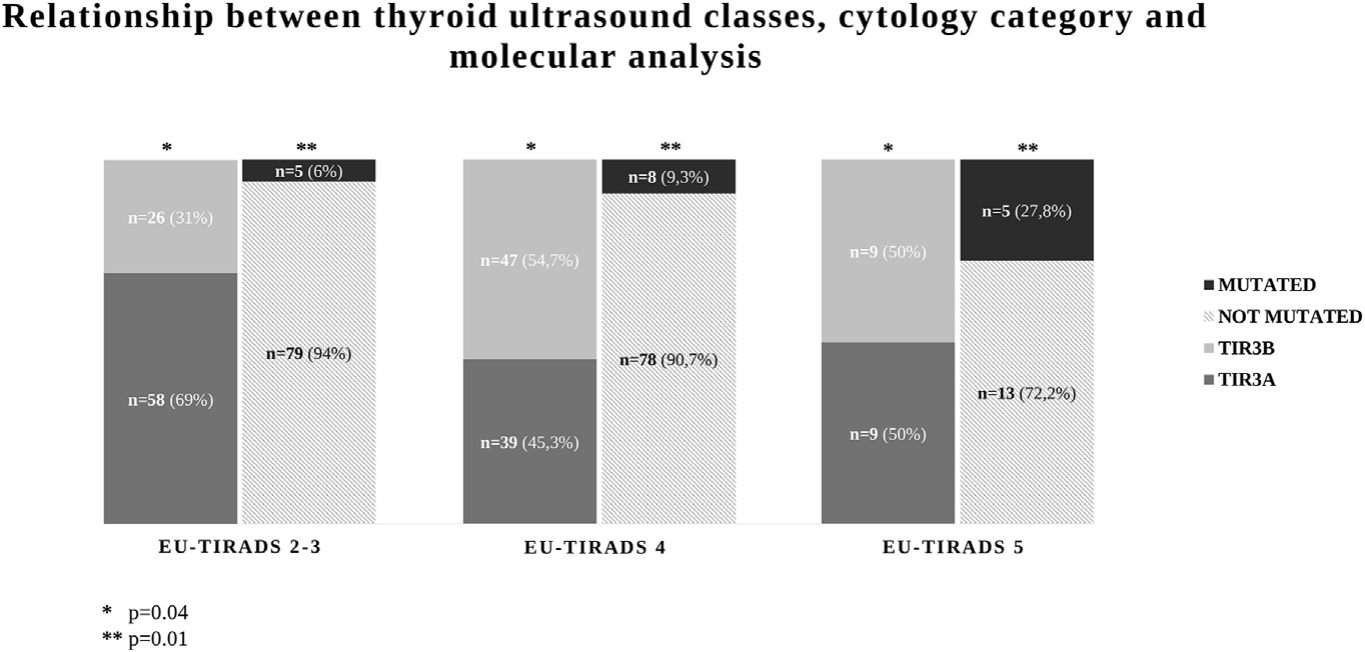
Relationship between thyroid ultrasound and cytological categories and seven-gene panel results in 188 indeterminate thyroid nodules (TIR3).

A significant correlation between EU-TIRADS categories and both cytological and molecular results was observed. In detail, TIR3B cytology was more frequent in EU-TIRADS 4 and 5 nodules (54.7 and 50%, respectively) than in EU-TIRADS 2–3 nodules (31%, *p* = 0.04). Similarly, the rate of nodules with a mutation increased with increasing of US risk class (6.0% in EU-TIRADS 2–3, 9.3% in EU-TIRADS 4 and 27.8% in EUTIRAD 5, *p* = 0.01) (**Figure 1**). In addition, we observed a significant correlation between US score and the seven-gene panel results in TIR3A (3.4% in EU-TIRADS 2–3, 2.5% in EU-TIRADS 4 and 33.3% in EUTIRAD 5, *p* = 0.0009), whereas no association was observed in TIR3B thyroid nodules (11.5% in EU-TIRADS 2–3, 14.8% in EU-TIRADS 4 and 22.2% in EUTIRAD 5, *p* = 0.73). The same results were observed when test for trend was used in both TIR3A and TIR3B nodules (p = 0.01 and p = 0.3, respectively).

### Surgical Series (n = 73)

Surgical treatment was performed in 73/188 (38.8%) indeterminate thyroid nodules. Among thyroid nodules submitted to surgery, final histology was benign in 44/73 (60.2%), while differentiated thyroid carcinoma was diagnosed in 29/73 (30.8%) thyroid nodules (**Table 2**). Among patients with thyroid carcinoma, we observed three cases of papillary thyroid cancer (PTC) in TIR3A group and 22 cases of PTC in TIR3B group. Two follicular thyroid cancers (FTCs) and two non-invasive follicular thyroid neoplasms with papillary like nuclear features (*NIFTP*) were also found in the TIR3B group. No association was observed with gender (*p* = 0.8), age at diagnosis (*p* = 0.1), nodular diameter (*p* = 0.8) and multinodular/uninodular goiter (*p* = 0.8) (**Table 2**). A significant correlation between cytological and histological diagnosis was observed (p < 0.001). As expected, the malignancy rate was significantly higher in TIR3B nodules (26/42, 66.6%) compared to TIR3A nodules (3/31, 9.7%) (*p* < 0.0001). A comparative analysis was performed between ultrasound risk class and histological results. The malignancy rate was lower in EU-TIRADS 2–3 [29.4% (10/34)] compared to EU-TIRADS 4–5 thyroid nodules [48.7% (19/39)], but this difference was not statistically significant (*p* = 0.10). A significant correlation was also observed between histological diagnoses and the results of the seven-gene panel. The malignant rate was 66.6% (12/18) in nodules with a mutation and 30.9% (17/55) in non-mutated nodules (*p* = 0.01).

**Table 2:** Demographic and clinical-pathological features in patients with indeterminate thyroid nodules submitted to surgery (n = 73).

Parameters	Malignant histology (n = 29)	Benign histology (n = 44)	*p*
**Gender: n (%)**			0.8**
Male	8 (27.6)	11 (25)	
Female	21(72.4)	33 (75)	
**Age at diagnosis (years)**			0.1*
Mean ± SD	49.2 ± 14.6	53.9 ± 13.4	
Range	18–81	20–79	
Median	52	55	
**Baseline Nodule diameter (mm)**			0.8*
Mean ± SD	31.5 ± 17.5	29.1 ± 12.6	
Range	9–82	8–58	
Median	28	29	
**Type of goiter n (%)**			0.8**
Multinodular goiter	17 (41.4)	24 (58.6)	
Uninodular goiter	12 (37.5)	20 (62.5)	
**Cytology classification**			<0.001**
TIR 3A	3 (9.7)	28 (90.3)	
TIR 3B	26 (66.6)	16 (33.4)	
**Ecographic score: n (%)**			0.10**
EUTIRADS 2-3	10 (29.4)	24 (70.6)	
EUTIRADS 4-5	19 (48.7)	20 (50.3)	
**Seven-gene panel**			0.01**
positive	12 (66.6)	6 (33.4)	
negative	17 (30.9)	38 (69.1)	
**Type of molecular alteration**			0.40******
BRAF (n = 2)	1 (50%)	1 (50%)	
RAS (n = 10)	8 (80%)	2 (10%)	
RET/PTC (n = 6)	3 (50%)	3 (50%)	

*By Mann–Whitney U test; **By X^2^ test. In bold significant p values (<0.05).

### US and Molecular Features as Criteria for Decision Making in TIR3A Nodules

**Figure 2:**
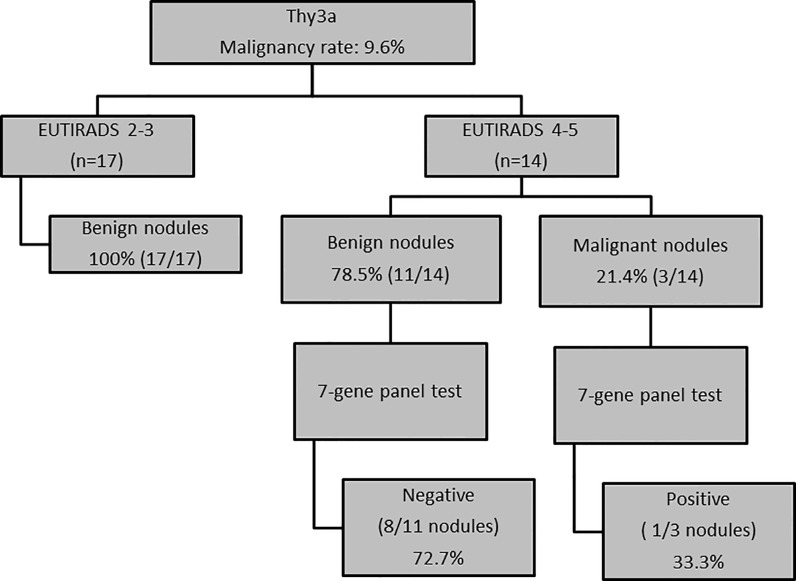
US and molecular features as criteria for decision-making in TIR3A nodules (n = 31 nodules).

Using only US score as criterion for decision-making in the TIR3A subgroup of indeterminate nodules, we correctly classified 20/31 (64.5%) of thyroid nodules. In detail, 100% of low risk US nodules were benign at histology, while the rate of malignancy in high risk US nodules was only 21.4%, with a trend toward significance (*p* = 0.08). In order to improve our ability in identifying benign nodules, we applied a multiple test using a series approach. Specifically, the second test (seven-gene panel test) was performed in the subgroup of US high-risk nodules (n = 14) in which, despite high risk US features, only 21.4% of thyroid nodules were malignant at histology. The seven-gene panel test analysis performed in the TIR3A nodule subgroup allowed us to correctly identify 73% of benign nodules and 33% of malignant nodules. Finally, using a multiple test with a series approach, 26/31 (84%) of thyroid nodules were correctly classified. Five out of 31 (16%) TIR3A nodules were not correctly identified. Among them, 2/31 (6.4%) were false negative of the seven-gene panels and very positive of US score while 3/31 (9.6%) were false positive of both US score and seven-gene panel (EU-TIRADS 4 and 5 nodules with a mutation) (**Figure 2**).

### US and Molecular Features as Criteria for Decision Making in TIR3B Nodules

**Figure 3:**
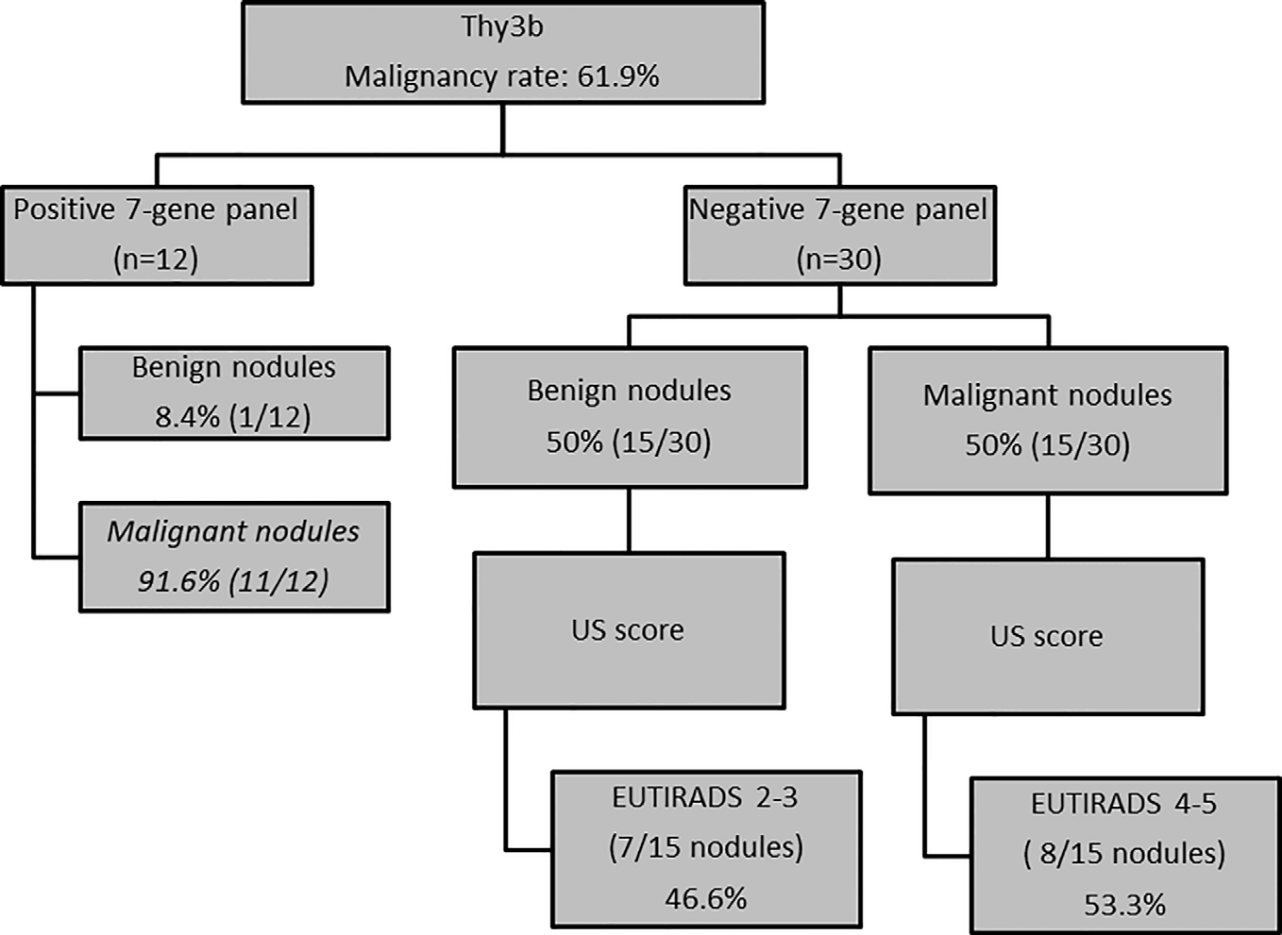
US and molecular features as criteria for decision-making in TIR3B nodules (n = 42 nodules).

Using only the seven-gene panel as criterion for decision-making in TIR3B nodules, we correctly classified 26/42 (61.9%) indeterminate nodules. In detail, all but one of the nodules with a mutation were malignant at histology (91.6%) while only 50% of non-mutated nodules were benign at histology (*p* = 0.01). In order to improve our ability in identifying malignant nodules, we applied a multiple test using a series approach. Specifically, the second test (US score) was evaluated in the subgroup of non-mutated thyroid nodules (n = 30) in which, despite the seven-gene panel, was negative, 50% of nodules showed malignancy at histology. Based on the results of US analysis, only 53% of malignant nodules had a high risk US features while the remaining malignant nodules were classified at low US risk of malignancy. Finally, using a multiple test with a series approach, only a slight improvement of the classification of thyroid nodules was observed [28/42 (66.6%)] (**Figure 3**).

## Discussion

Cytologically indeterminate thyroid nodules currently represent a challenge for clinical decision-making. Since the overall rate of malignancy is low, a tool that helps to rule out thyroid cancer preoperatively might reduce the number of diagnostic surgeries. To spare patients from unnecessary surgery, molecular testing as a diagnostic adjunct to FNA cytopathology were developed (21, 22), and the European Thyroid Associations have endorsed this recommendation, thereby underlining the importance of molecular tests on thyroid FNAC (3). Moreover, in the recent years, ultrasound (US) has increasingly gained a central role in the evaluation of thyroid nodules that are eligible for FNAC (23). Recent studies have addressed the role of US scores in indeterminate thyroid nodules (24–26), and some authors reported that the US patterns should not only select the nodules for biopsy but also triage cytologically indeterminate lesions for US follow-up, molecular analysis or surgery (27). Since at our Institute a seven-gene panel was routinely performed in indeterminate thyroid nodules, we aimed to verify the utility of US score in selecting indeterminate nodules for molecular analysis. We found a significant correlation between US score and mutational status (*p* = 0.01). We also found a significant correlation between US score and cytological sub-classification of indeterminate thyroid nodules (*p* = 0.04) suggesting that US patterns are significantly associated with both mutational status and distinct cytological profiles.

We, also, compared histological results with US features in a subgroup of indeterminate thyroid nodules submitted to surgery (n = 73). The cancer rate was higher in US high risk nodules (48.7 *versus* 29.9% in low-risk US nodules) but this difference fell just short of statistical significance (*p* = 0.10), probably due to the small cohort of indeterminate thyroid nodules submitted to surgery. Nevertheless, the probability to have benign nodules was very high (100% in our cohort) in the subgroup of low risk indeterminate cytology (TIR3A) classified at low risk US score, suggesting the possibility of a conservative approach without additional test in this subgroup of indeterminate nodules. Conversely, US risk stratification system seems to have low ability to correctly identify malignant nodules in both TIR3A and TIR3B nodules. Similarly to our results, the distribution of histological diagnoses in indeterminate thyroid nodules was significantly different within different US patterns in the study of Valderrabbano et al. (15). The rates of malignancy for the very low, low/intermediate, high, and non-ATA patterns were 0, 19, 56, and 36%, respectively, and were all significantly different. Nevertheless, the majority of low risk US nodules were benign at histology while in the intermediate/high risk US nodules thyroid cancer was found in only 50% of them, suggesting that US features alone are not able to guide the decision-making process in indeterminate thyroid nodules with high risk US features (15). As expected, a significant correlation was also observed between seven-gene panel results and histological results (*p* = 0.01). It has been previously reported that the seven-gene panel provides high PPV results and can “rule-in” cancer (28). Therefore, a positive molecular result warrants a therapeutic surgery. We found a nodule mutated for BRAF with negative histology for thyroid cancer. However, it cannot be excluded that sporadically scattered malignant cells escaped microscopic observation although a recent meta-analysis reported some sporadic cases (1.2%) of indeterminate thyroid nodules with BRAF mutation associated with benign histology (29). At the same way, the same papers reported the presence of RET/PTC rearrangements in 5% of benign thyroid nodules and several cases of Hashimoto’s thyroiditis (30, 31). In the present study molecular markers in the cytological samples correctly identified thyroid cancers in 66.7% of cases; however, this percentage varied significantly from 16.6% in TIR3A to 91.7% in TIR3B nodules, suggesting that in the presence of positive seven-gene panel the probability of cancer is significantly higher in cytological high risk indeterminate thyroid nodules. Moreover, because of its low sensitivity, the seven-gene panel cannot “rule-out” cancer, and a negative molecular result cannot avoid diagnostic surgery, limiting their cost-effectiveness (7). Indeed, in this study benign nodules were correctly identified in 70% of cases even though this percentage varied significantly from 50% in TIR3B to 92% in TIR3A nodules, confirming the ability of the seven-gene panel to rule-out thyroid cancer at least in low risk indeterminate thyroid nodules (TIR3A) (data not shown). Finally, we verified whether the combination of US score and the seven-gene panel results was able to improve the pre-surgical diagnosis of indeterminate thyroid nodules using a series approach of the two different tests. In TIR3A nodules at low US risk features, the combination of US score and the seven-gene panel did not significantly change the decision-making. Indeed, in this subgroup of nodules the cancer risk was very low, regardless of the mutational status. Conversely, in the subgroup of high US risk in which the cancer rate was low (around 20%), the seven-gene panel was helpful in correctly identifying benign nodules (73% of cases). Instead, the combination of US score and seven-gene panel did not improve the pre-surgical diagnosis in high risk indeterminate nodules (TIR3B). Specifically, the rate of thyroid cancer is not correlated with US risk class, and the rate of the seven-gene panel false negative considerably limits the clinical applicability of seven-gene panels in high risk indeterminate nodules (TIR3B). To our knowledge, only one study evaluated the impact of US features, cytomorphology, and mutational testing in thyroid nodules (32). The authors concluded that both PPV and NPV were increased by the contribution of additional methodologies. Nevertheless, in contrast to our study, the authors included in the study group other cytological categories (such as TIR4 and TIR5 thyroid nodules) with different rates of malignancy, and this might explain the very high NPV and PPV of the combination of US and molecular features reported in this study (88 and 97%, respectively). Indeed, in the subgroup of 42 indeterminate thyroid nodules (TIR3), similarly to our results, all TIR3A nodules with low-risk US features were benign at histology, supporting our hypothesis that surgery or additional test may be avoided in this subgroup of thyroid nodules. On the contrary, the combination of US features and molecular results was not helpful to improve the diagnosis in US high-risk TIR3A and TIR3B nodules, probably due to the small number of cases evaluated (32).

The main limitation of our study was the small size of our cohort mainly due to the difficulty of retrieving thyroid US images and histological results of all patients. The other limitation was not having access to the NGS multi-gene panel such as Thyroseq v3 which allows the analysis of more than hundred genes and detection of different classes of genetic mutations. Finally, due to the retrospective design, patient selection bias may have occurred in our study, especially in the subgroup of patients submitted to surgery. Indeed, after cytological and molecular results, the decision-making process was defined by the care provider of the patients. Therefore, we acknowledge that the results could be different in other Centers. For this reason we believe that, although promising, our results should be confirmed in a prospective study including a larger series of indeterminate thyroid nodules. The strengths of our study are as follows. First, the confirmation of indeterminate cytology by a second FNAC in all patients included in the study. Second, to the best of our knowledge, this is the first study to evaluate the performance of US score and the seven-gene panel exclusively on indeterminate thyroid nodules using a series approach.

In conclusion, US score seems able to correctly discriminate between benign TIR3A nodules in which a conservative approach may be valid and those in which additional test, such as molecular test, may be indicated. On the contrary, in TIR3B nodules, the US risk stratification as well as the seven-gene panel, appears to be of little utility because the risk of thyroid cancer remains high regardless of US score and mutational status.

## Data Availability Statement

The raw data supporting the conclusions of this article will be made available by the authors, without undue reservation.

## Ethics Statement

The studies involving human participants were reviewed and approved by the Comitato Etico regione Toscana-Area Vasta sud-est. The patients/participants provided their written informed consent to participate in this study.

## Author Contributions

MC and MGC contributed to the conception, design, and interpretation of the project. SC, AnS, and AlS contributed to the experimental part and data analysis of the project. TP, CC, FM, RF, and AC contributed to drafting or revising intellectual content of the manuscript. MC and MGC had primary responsibility for final content. All authors contributed to the article and approved the submitted version.

## Conflict of Interest

The authors declare that the research was conducted in the absence of any commercial or financial relationships that could be construed as a potential conflict of interest.
